# Functional Characteristics of Caffeoyl Shikimate Esterase in *Larix Kaempferi* and Monolignol Biosynthesis in Gymnosperms

**DOI:** 10.3390/ijms20236071

**Published:** 2019-12-02

**Authors:** Xuechun Wang, Nan Chao, Meng Zhang, Xiangning Jiang, Ying Gai

**Affiliations:** 1 College of Biological Sciences and Biotechnology, Beijing Forestry University, Beijing 100083, China; wangxuechun0717@126.com (X.W.); chaonan1989@126.com (N.C.); zhangmeng199628@163.com (M.Z.); jiangxn@bjfu.edu.cn (X.J.); 2 National Engineering Laboratory for Tree Breeding, the Tree and Ornamental Plant Breeding and Biotechnology Laboratory of Chinese Forestry Administration, Beijing 100083, China

**Keywords:** caffeoyl shikimate esterase (CSE), gymnosperms, *Larix kaempferi*, monolignol, ER-resident

## Abstract

Caffeoyl shikimate esterase (CSE) has been reported to be involved in lignin biosynthesis; however, studies of CSE in gymnosperms are lacking. In this study, *CSE* was successfully cloned from *Larix kaempferi* (*LkCSE*) based on *Larix laricina* transcriptome screening. LkCSE was likely to have catalytic activity based on homologous sequence alignment and phylogenetic analyses of CSEs from different species. In vitro assays with the recombinant enzyme validated the catalytic activity of LkCSE, indicating its function in converting caffeoyl shikimate into caffeate and shikimate. Additionally, the optimum reaction pH and temperature of LkCSE were determined to be 6.0 and 30 °C, respectively. The values of *K_m_* and *V_max_* of CSE for caffeoyl shikimate were 98.11 μM and 14.44 nM min^−1^, respectively. Moreover, *LkCSE* was observed to have tissue expression specificity and was abundantly expressed in stems and leaves, especially stems, which was 50 times higher than the expression levels of roots. Lastly, translational fusion assays using LkCSE fused with green fluorescent proteins (GFP) in tobacco leaves indicated that LkCSE was localized in the plasma membrane and endoplasmic reticulum (ER). These results revealed that CSE clearly functions in gymnosperms and it is possible for LkCSE to interact with other ER-resident proteins and regulate mass flux in the monolignol biosynthesis pathway.

## 1. Introduction

Lignin is one of the most abundant biomass components in plants and has been extensively studied for its vital biological functions (e.g., mechanical support, water retention, and barrier against infection), as well as its limitations in the conversion efficiency of lignocellulosic biomass to ethanol [1,2,3,4,5]. The ability to produce trees with less lignin or more easily degradable lignin, while simultaneously maintaining normal growth, would reduce the high processing costs and carbon footprint of the manufacturing process of paper, biofuels, and chemicals [6,7,8,9,10]. Lignin is a natural aromatic polymer generated by the radical coupling of monolignols (hydroxycinnamyl alcohols), including coniferyl, sinapyl alcohols, and minor amounts of *p*-coumaryl alcohol, which are the main building blocks of lignin [11]. The phenylpropanoid pathway has been reported to be the biosynthetic pathway for monolignols and has been continuously revised over time, as changing the mass flux in the lignin biosynthesis pathway is the most obvious approach for modifying lignin content [12,13,14].

*P*-coumarate 3-hydroxylase (C3’H) is associated with hydroxylcinnamoyl-CoA shikimate/quinate hydroxycinnamoyl transferase (HCT), and diverts the pathway away from H-lignin toward G- and S-lignin [15,16,17,18]. In this flux diversion process, HCT catalyzes in two steps and the second reaction leads to the conversion of caffeate esters to caffeoyl-CoA. Caffeate esters (mainly caffeoyl shikimate) are important intermediates in lignin biosynthesis, and the discovery of caffeoyl shikimate esterase (CSE) revises our understanding of the phenylpropanoid pathway [19]. Enzymatic analyses of recombinant CSE proteins in *Arabidopsis thaliana* indicated that this enzyme can hydrolyze caffeoyl shikimate into caffeate. Further studies on *A. thaliana* CSE mutants revealed that lignin content decreased with increasing levels of *p*-hydroxyphenyl units, leading to an accumulation of intermediate caffeoyl shikimate [19]. CSE together with 4-coumarate: CoA ligase (4CL) bypasses the second HCT reaction and directs the flux away from H-lignin toward G- and S-lignin. However, a study reported that secondary differentiating xylem protein extracts of *Populus trichocarpa*, *Eucalyptus grandis*, and the stems of *Panicum virgatum* and *Oryza sativa* did not show CSE activity [20]. A separate study on *P. virgatum* indicated that CSE is likely to function as reported in *Arabidopsis* and bypass the second HCT reaction [21]. Similarly, in a recent study, detectable CSE activity was found in the crude extraction of *P. virgatum*, as well as purified recombinant CSE proteins in *Medicago truncatula* and *Populus deltoids* [22]. Additionally, CSE loss-of-function in *M. truncatula* resulted in severe dwarfing, reduction in lignin content, and preferential accumulation of hydroxyphenyl units, which were similar to the study on the *Arabidopsis* CSE mutant [22]. Increasing saccharification was also observed when CSEs were silenced in poplar [14]. However, reactions catalyzed by CSE may not be essential for lignification in all plant species, as crude protein extracts are found in the stems of *Brachypodium distachyon* and *Zea mays*, which have no orthologs of the currently characterized *CSE* genes and exhibit only a weak esterase activity with caffeoyl shikimate [22].

Although several studies have reported CSE activity in different plant species, little is known about whether CSE functions in gymnosperms. Moreover, it has been reported that CSE does not have orthologs in some plants, such as *B. distachyon* and *Z. mays*, and that monolignol biosynthesis and lignin composition in gymnosperms function somewhat differently from those in angiosperms [22,23]. Thus, it is important to validate whether the bypass route catalyzed by CSE also functions in gymnosperms. In this study, we identified and cloned an ortholog of *CSE* in *Larix kaempferi* (*LkCSE*) based on the published *Larix laricina* FK-6-B transcriptome (SRX4092599). Further analyses of *LkCSE* revealed its function in monolignol biosynthesis of *L. kaempferi* based on its subcellular location, expression profile, and enzymatic assay, which provides insights for the function of CSEs in gymnosperms.

## 2. Results

### 2.1. Identification and Cloning of Caffeoyl Shikimate Esterase (CSE) Ortholog in Larix Kaempferi

Because CSE in gymnosperms has not been reported, and the transcriptome and genome information of *L. kaempferi* is not available, the *L. laricina* FK-6-B transcriptome (SRX4092599) was adopted for CSE ortholog identification. The transcript (TRINITY_DN47728_c0_g1_i1) was identified as the CSE ortholog transcript in *L. laricina* with distinct high scores, query cover > 90%, identity > 50%, and a low E-value (E-100). The *LkCSE* (accession No: MK211161) in *L. kaempferi* was obtained based on the *CSE* sequence information of *L. laricina*. *LkCSE* encoded 193 amino acids and had a putative molecular weight of approximately 35.95 kDa.

### 2.2. LkCSE —A CSE Ortholog with Conserved Motifs

**Figure 1:**
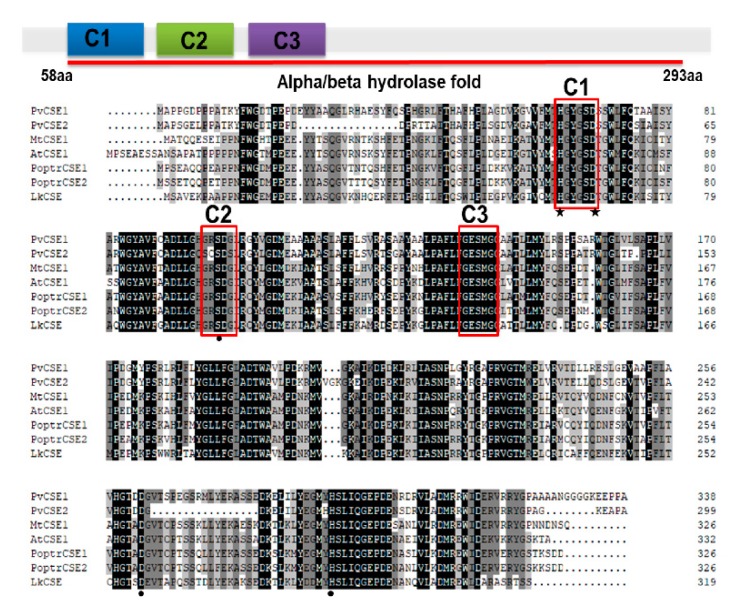
Alignment of LkCSE (caffeoyl shikimate esterase in *Larix kaempferi)* and validated CSE sequences. Conserved and functional motifs are indicated by a red box, including an acyltransferase motif (C1: HX_4_D) and two lipase motifs (C2 and C3: GXSXG). Key amino acids involved in the enzymatic activity are marked with black stars (acyltransferase activity) or circles (catalytic triad).

**Figure 2:**
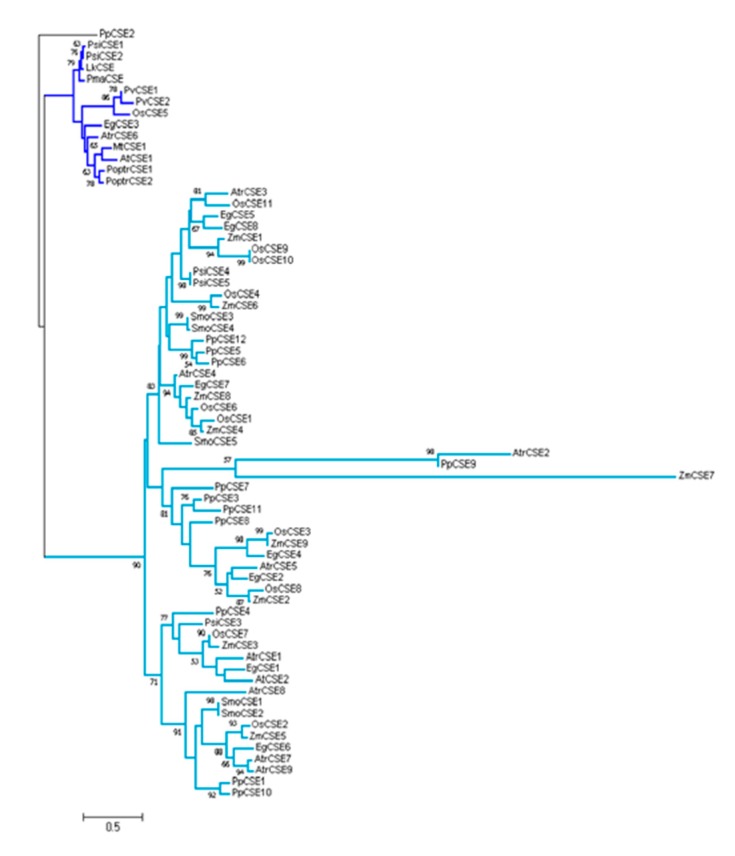
Phylogenetic tree of screened CSE and CSE-like protein sequences from *pinus* and other species. Verified CSEs are marked in dark blue. PpCSE2 (*Physcomitrella patens*) was used as the root.

Six CSEs and LkCSE were aligned. Conserved motifs, acyltransferase (HX_4_D) and two hydrolase (GXSXG) motifs, were identified in these putative proteins [24,25]. Clearly, LkCSE possesses all these conserved motifs with high sequence identity with the six CSEs (Figure 1). Further phylogenetic analyses were performed on the six CSEs and genome-wide screened putative CSEs in PLAZA (Figure 2). The GXSXG motifs in these putative proteins were highly conserved, corresponding to the lipase/esterase superfamily. LkCSE clustered with the verified CSEs from fern, dicots, and monocots, further indicating that LkCSE is a CSE ortholog. A CSE ortholog, *OsCSE5*, was identified in *O. sativa*, while none was detected in *Z. mays.* In fact, in most monocots, such as *B. distachyon* and *Z. mays*, CSE was excluded, while *O. sativa* seemed to be an exception [22]. These results suggest that CSEs exist in gymnosperms and are lost in major monocots.

### 2.3. Subcellular Localization of LkCSE

**Figure 3:**
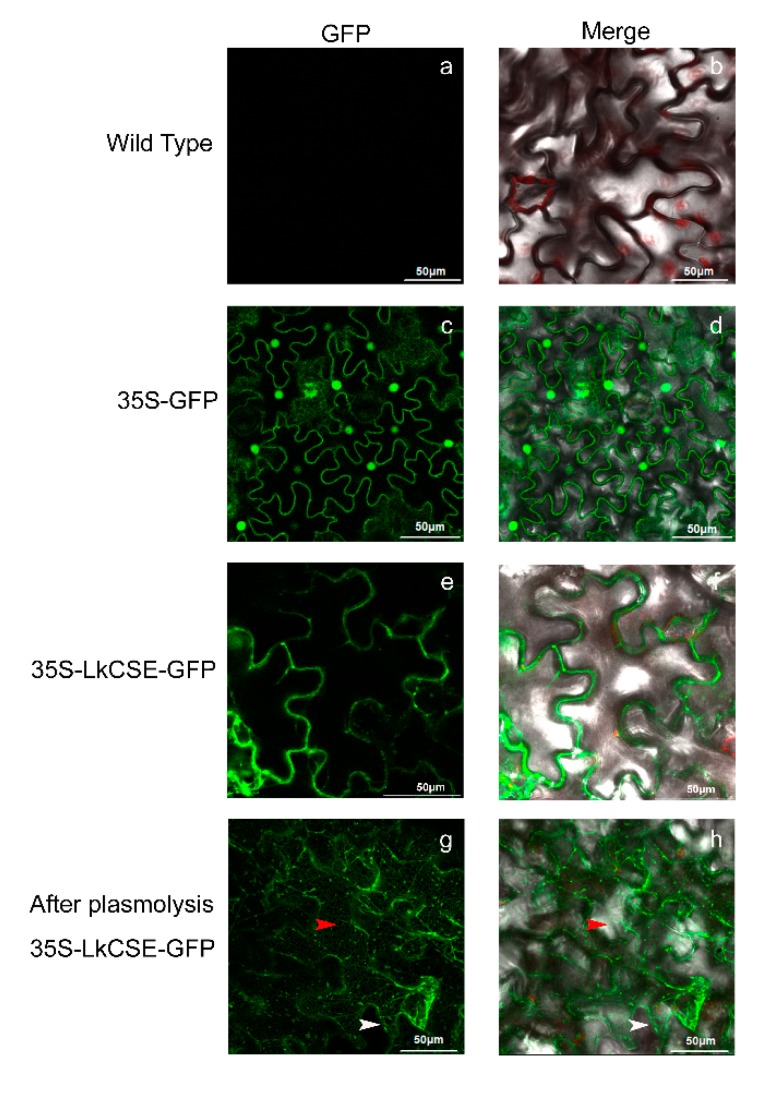
Subcellular localization of LkCSE in transformed tobacco leaves: (**a**,**b**) wild-type; (**c**,**d**) 35S-GFP; (**e**,**f**) 35S-LkCSE-GFP (green fluorescent protein); and (**g**,**h**) 35S-LkCSE-GFP plasmolysis. The red arrow and white arrow indicate the ER and plasma membrane, respectively.

To explore the subcellular localization of LkCSE, 35S-LkCSE-GFP (green fluorescent protein), and 35S-GFP were constructed with the pBI121 vector and transferred into the *Agrobacterium tumefaciens* strain, *GV3101*, then transformed to tobacco leaves. Using a confocal microscope to compare the control wild-type (Figure 3a,b) and 35S-GFP (Figure 3c,d), LkCSE with fused GFP was observed in the plasma membrane and endoplasmic reticulum (ER) (Figure 3e,f). Following plasmolysis, green fluorescence retracted from the plasma membrane with some signals appearing in the ER of 35S-LkCSE-GFP transformed tobacco leaves (Figure 3g,h). This result corresponds with the subcellular localization of AtCSE (At1g52760) (*A. thaliana*), which was first reported as lysophospholipase 2 (lysoPL2) [24]. The subcellular localization of L-phenylalanine ammonia-lyase (PAL), Cinnamate 4-hydroxylase (C4H), C3’H, and ferulate 5-hydroxylase (F5H) were reported to be ER-resident proteins that assemble multi-enzyme complexes, including Ptr4CL3/4CL5 (*P. trichocarpa*) and PtrC4H1/C4H2/C3’H3; PAL and HCT involved in monolignol biosynthesis were also reported [26]. It is possible that CSE can also assemble with ER-resident proteins as complexes to control the bypass of monolignol biosynthesis.

### 2.4. Tissue Expression Profile of LkCSE Revealed Tissue Specificity

**Figure 4:**
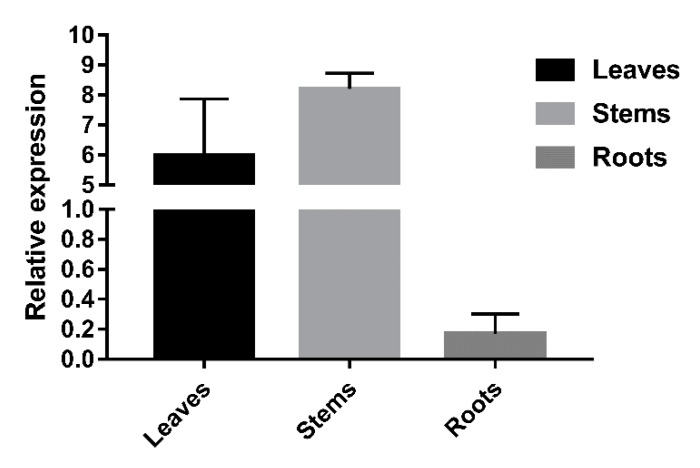
The LkCSE expression profile in different tissues from *L. kaempferi*. Total RNA was extracted from the leaves, roots, and stems of *L. kaempferi*. *Elongation factor-1 alpha 1* (*EF1A1*) was used as an internal reference gene. Three replicates were performed in parallel and formed the standard error.

The real-time quantitative PCR (RT-qPCR) results revealed that *LkCSE* was expressed in the stems and leaves. Based on the relative expression, which was normalized using *elongation factor-1 alpha 1* gene (*EF1A1*) (accession No: JX157845), the expression levels of *LkCSE* in the roots were very low compared to the stems. In contrast, the highest expression level of *LkCSE* was in the stems with roughly 50 times more than the expression level of roots (Figure 4). Key genes of monolignol biosynthesis prefer expression in tissues involved in lignification, especially xylem [27]. The high expression of *LkCSE* in the stems indicated that it plays a role in lignification. Shikimate, the catalytic product of CSE, was found to be a major component of pine needle organic acids and was abundantly present in needles [28,29], so we suggested that CSE may have functions in producing shikimic acid, thereby highly expressed in leaves.

### 2.5. LkCSE Converts Caffeoyl Shikimate to Caffeate and Shikimate

**Figure 5:**
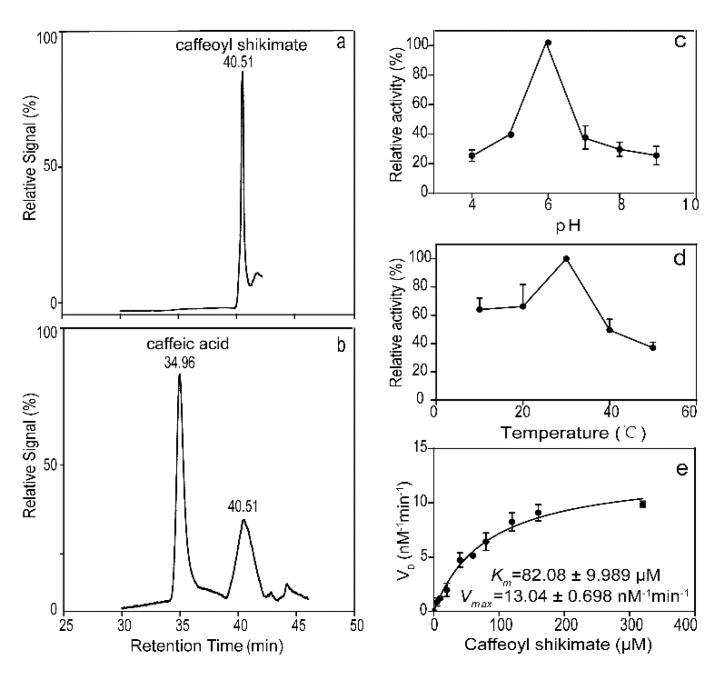
Enzymatic assays of LkCSE. (**a**) Control; (**b**) Purified LkCSE; (**c**) The optimum reaction pH and (**d**) temperature for LkCSE; (**e**) Enzyme kinetic was obtained by Graphpad Prism 7.0 (GraphPad, San Diego, CA, USA) with Michaelis–Menten enzyme kinetics curve. All samples were determined by measuring caffeic acid using high-performance liquid chromatography–mass spectrometry with sinapic acids as internal standards. Three replicates were created for each sample in parallel and formed the standard error.

CSE activity was detected using high-performance liquid chromatography–mass spectrometry (HPLC-MS) and it was found that LkCSE had detectable activity against caffeoyl shikimate (Figure 5a,b). The optimum pH of LkCSE was 6.0 (Figure 5c), while the optimum temperature was 30 °C (Figure 5d). The values of *K_m_* and *V_max_* of CSE for caffeoyl shikimate were 82.08 μM and 13.04 nM min^−1^ per microgram protein, respectively (Figure 5e), and *K_cat_/K_m_* = 0.0104 s^−1^μM^−1^. LkCSE affinity for caffeoyl shikimate is higher than AtCSE. Catalytic efficiency for caffeoyl shikimate of LkCSE is nearly three-fold higher than that of AtCSE (*K_cat_/K_m_* = 0.00357 s^−1^μM^−1^) [19]. These results indicate that LkCSE may have the ability to change the mass flux of the monolignol biosynthesis pathway in *L. kaempferi.*

## 3. Discussion

### 3.1. CSE Functions in Gymnosperms

CSE has been identified as a key enzyme involved in monolignol biosynthesis of *Arabidopsis*. However, there is some debate and contradicting evidence of the function of CSE in *P. virgatum*, which perpetuates confusion of CSE’s function in plants [20,22]. Moreover, CSE was not even detected in some plants, such as *B. distachyon* and *Z. mays* [22]. This information suggests that CSE may not be a prevailing component in all plants. Gymnosperms have been reported to possess distinct components of monolignols with minor or no S-monolignol, and are thought to lose F5H in the pathway [23]. Therefore, whether a CSE ortholog in gymnosperms has a function in monolignol biosynthesis remains to be determined. Cloning and biochemical assays of *LkCSE* revealed that CSE can change the mass flux of the monolignol biosynthesis pathway by converting caffeoyl shikimate to caffeate and shikimate in *L. kaempferi.* This suggests that CSE may have a function in gymnosperms. Given the loss of CSE in several monocots, it is possible that ancestral CSE appears before the divergence of angiosperms and gymnosperms and is lost during evolution as adaptations for lignin components developed, especially for most monocots.

### 3.2. ER Resident CSE Provides Insight for Monolignol Biosynthesis Flux Regulation

CSE and other ER-resident proteins, such as C3’H and C4H, can regulate the flux of monolignol biosynthesis. Recent studies have revealed that proteins involved in monolignol biosynthesis could form complexes and play certain functional roles, such as cinnamoyl alcohol dehydrogenase 1 (CAD1) and cinnamoyl-CoA reductase 2 (CCR2), Ptr4CL3/4CL5, and PtrC4H1/C4H2/C3’H3 [30,31]. As ER-resident proteins, CSEs are likely to interact with other proteins, such C3’H, C4H, and F5H, and assemble complexes to efficiently regulate the mass flux from H-lignin toward G- and S-lignin. Moreover, a previous study reported that acyl-CoA-binding protein 2 (ACBP2) can bind with CSE and lysophosphatidylcholine (lysoPC) to promote tolerance to cadmium-induced oxidative stress in *Arabidopsis* [32]. However, whether this interaction could affect monolignol biosynthesis is unknown. Thus, as an ER-resident, CSE with its interactors, consisting of ER-resident proteins and HCT or 4CL, may control the switch for the bypass of monolignol biosynthesis.

## 4. Materials and Methods

### 4.1. Plant Materials

Two-year-old *L. kaempferi* was cultivated in Liaoning province, China. Stems (including xylem) were collected at 10 cm below the apex of the plant. All of the axial roots and leaves were collected. The samples were obtained at 8 a.m. on September 23, 2018, and immediately frozen in liquid nitrogen and stored at −80 °C for future use. Three biological replicates were performed for each sample.

### 4.2. Transcriptome Based Identification of CSE Genes in L. Kaempferi

Seed sequences, *AtCSE1*, *PvCSE1* (*P. virgatum*), *PoptrCSE1* (*P. trichocarpa*), *MtCSE* (*M. truncatula*), and *PmaCSE* (*Pinus massoniana*), were retrieved from the published literature and NCBI database [14,19,22]. Using these CSEs as query sequences, Blastn and tBlastn were locally performed to identify CSE orthologs against the published *L.laricina* FK-6-B transcriptome(SRX4092599) (Table S1: Blastp screening for LkCSEs with larix_laricina_fk-6-b.transcriptome). Based on this sequence information, primer pairs were designed and the CSE ortholog, *LkCSE* (MK211161), was cloned from stems of *L. kaempferi* (Table S2: Information on *LkCSE* primers).

### 4.3. CSE Sequence Alignments and Phylogenetic Analyses

Six validated CSE sequences including *AtCSE1, PvCSE1, PvCSE2, PoptrCSE1, PoptrCSE2*, and *MtCSE* were collected. The alignment of *LkCSE* and the 6 CSEs was performed using DNAMAN v8.0 (Lynnon Corporation, Vandreuil, QC, Canada) and CLUSTAL W assembled in Mega v6.0 with default parameters [33]. Hydrolase (GXSXG) and acyltransferase (HX_4_D) motifs were identified by submitting sequences to Pfam (http://pfam.janelia.org). A phylogenetic tree was generated using Mega v6.0 with the maximum-likelihood method, the substitute JTT model, and G + I rates among site models. The reliability of internal branches was assessed by using 500 bootstrap replicates and marked above nodes greater than 50. The putative CSE sequences used for phylogenetic analyses were obtained from the published literature and the PLAZA v3.0 platform (https://bioinformatics.psb.ugent.be/plaza/). CSE sequences from the gymnosperms, *Picea sitchensis* and *Selaginella moellendorffii*, were retrieved using BlastP of the NCBI database. All CSEs and CSE homologs are available online (Table S3: Putative CSE information for phylogenetic analysis).

### 4.4. Subcellular Location of LkCSE

The pBI121 vector with the *LkCSE* and GFP fusion expression was constructed. Then, the recombinant plasmids were transferred into the *Agrobacterium tumefaciens* strain, *GV3101*, which was transferred into tobacco leaves via *Agrobacterium*-mediated transient transformation [34]. The tobacco leaves were cut into squares of 5 mm × 5 mm, and then plasmolysis was performed [35]. GFP fluorescence in plant leaves was observed using a Leica TCS SP8 confocal microscope (Leica Microsystems, Wetzlar, Germany).

### 4.5. LkCSE Expression Profile in Different Tissues

Total RNA was extracted from 100 mg leaves, roots and stems in *L. kaempferi*. Total RNA with 500 ng was reverse transcribed into cDNA using a reverse transcription kit (Aidlab, Beijing, China). RT-qPCR was performed using a 7500 Fast Real-Time PCR system (Applied Biosystems, Foster, CA, USA) with SYBR Premix Ex TaqTM (Aidlab, Beijing, China) and using *EF1A1* as an internal reference gene [36]. Three replicates were made for each tissue in parallel.

### 4.6. Purification of Recombinant LkCSE

*LkCSE* was cloned into pET28a (Novagen, Madison, WI, USA), which was transformed into the *Escherichia coli* strain BL21 (DE3). The recombinant strains were cultured in LB medium containing 500 mg/L kanamycin at 37 °C to OD_600_ = 0.6, and the protein expression was induced at 25 °C for 8 h by adding 0.4 mM isopropyl β-d-thiogalactoside (IPTG). The proteins were purified using Ni-NTA agarose (Qiagen, Hilden, Germany). The column was washed with wash buffer (50 mM Tris-Hcl pH 8.0, 300 mM Nacl, and 20 mM imidazole) and eluted with elution buffer (50 mM Tris-Hcl pH 8.0, 300 mM Nacl, and 150 mM imidazole).

### 4.7. High-performance Liquid Chromatography–mass Spectrometry Based Enzymatic Assays

Enzymatic reactions were performed following the methods outlined by Escamilla-Treviño et al. (2014) [21]. Purified LkCSE was incubated at 30 °C for 30 min with 100 mM NaPO_4_ buffer (pH = 7.5), 500 μM dithiothreitol, and 100 μM caffeoyl shikimate (ChemFaces, Wuhan, China) with a final volume of 100 μL, and boiled for 10 min as control. All samples, including the controls, were terminated by adding 10 μL of glacial acetic. Standard samples of substrates and products are in Figure S1: HPLC data of caffeoyl shikimate and caffeic acid. To explore the optimum reaction temperature, samples were incubated at various temperatures ranging from 10 °C to 50 °C. Multi-pH phosphate buffers ranging from 4.0 to 9.0 were adopted to explore the optimum reaction pH for CSE. Enzyme kinetics of the purified recombinant LkCSE were analyzed at 100 μL with 1 μg purified protein, 100 mM NaPO_4_ buffer (pH = 6.0), 500 μM dithiothreitol, and 0–320 μM caffeoyl shikimate. Kinetic parameters were obtained by Graphpad Prism 7.0 (GraphPad, San Diego, CA, USA) with a Michaelis–Menten enzyme kinetics curve. Reaction products with 10 ng/μL sinapic acids that were added as internal standards were injected into HPLC-MS (Agilent, Wilmington, DE, USA) with a reverse-phase C18 column and separated in a step gradient using 1‰ formic acid in water as solvent A and 0.1% formic acid in acetonitrile as solvent B to detect caffeic acid. Detailed information is available in Ha et al. (2016) [22].

## 5. Conclusions

Several studies have been conducted on CSE in angiosperms, while little is known about whether CSE functions in gymnosperms. A novel *CSE* gene from *L.kaempferi* (*LkCSE*) was cloned and investigated in this study. The phylogenetic analyses from different plant species indicated that CSE appears before the divergence of angiosperms and gymnosperms, but may get lost during evolution due to adaptation for the lignin component, especially for most monocots. The *LkCSE* had tissue expression specificity and was highly expressed in stems, indicating it plays a role in lignification. LkCSE was localized in the plasma membrane and endoplasmic reticulum and formed a complex with ER-resident proteins to regulate the bypass way of monolignol biosynthesis. LkCSE had detectable activity which converts caffeoyl shikimate to caffeate and shikimate. These results also suggest that LkCSE has functions in monolignol biosynthesis and provide insights for the function of CSEs in gymnosperms.

## Supplementary Materials

Supplementary Materials can be found at https://www.mdpi.com/1422-0067/20/23/6071/s1.

## Abbreviations

4CL	4-coumarate: CoA ligase
ACBP2	Acyl-CoA-binding protein 2
C3’H	P-coumarate 3-hydroxylase
C4H	Cinnamate 4-hydroxylase
CAD	Cinnamyl alcohol dehydrogenase
CCR	Cinnamoyl-CoA reductase
EF1A1	Elongation factor-1 alpha 1
ER	Endoplasmic reticulum
F5H	Ferulate 5-hydroxylase
GFP	Green fluorescent protein
HCT	Hydroxylcinnamoyl-CoA shikimate/quinate hydroxycinnamoyl transferase
LysoPC	Lysophosphatidylcholine
LysoPL2	Lysophospholipase 2
PAL	L-phenylalanine ammonia-lyase
