# Supplementary material for: Functional Characteristics of Caffeoyl Shikimate Esterase in Larix Kaempferi and Monolignol Biosynthesis in Gymnosperms
Source: Int J Mol Sci. 2019 Dec 2;20(23):6071. doi: 10.3390/ijms20236071 (PMC6929169; doi:10.3390/ijms20236071)
Supplement: Supplementary file 1 [file ijms-20-06071-s001.zip › ijms-639881-supplementary/Supplementary Material 4.docx]

**Supplementary Material 4**. HPLC data of caffeoyl shikimate and caffeic acid.


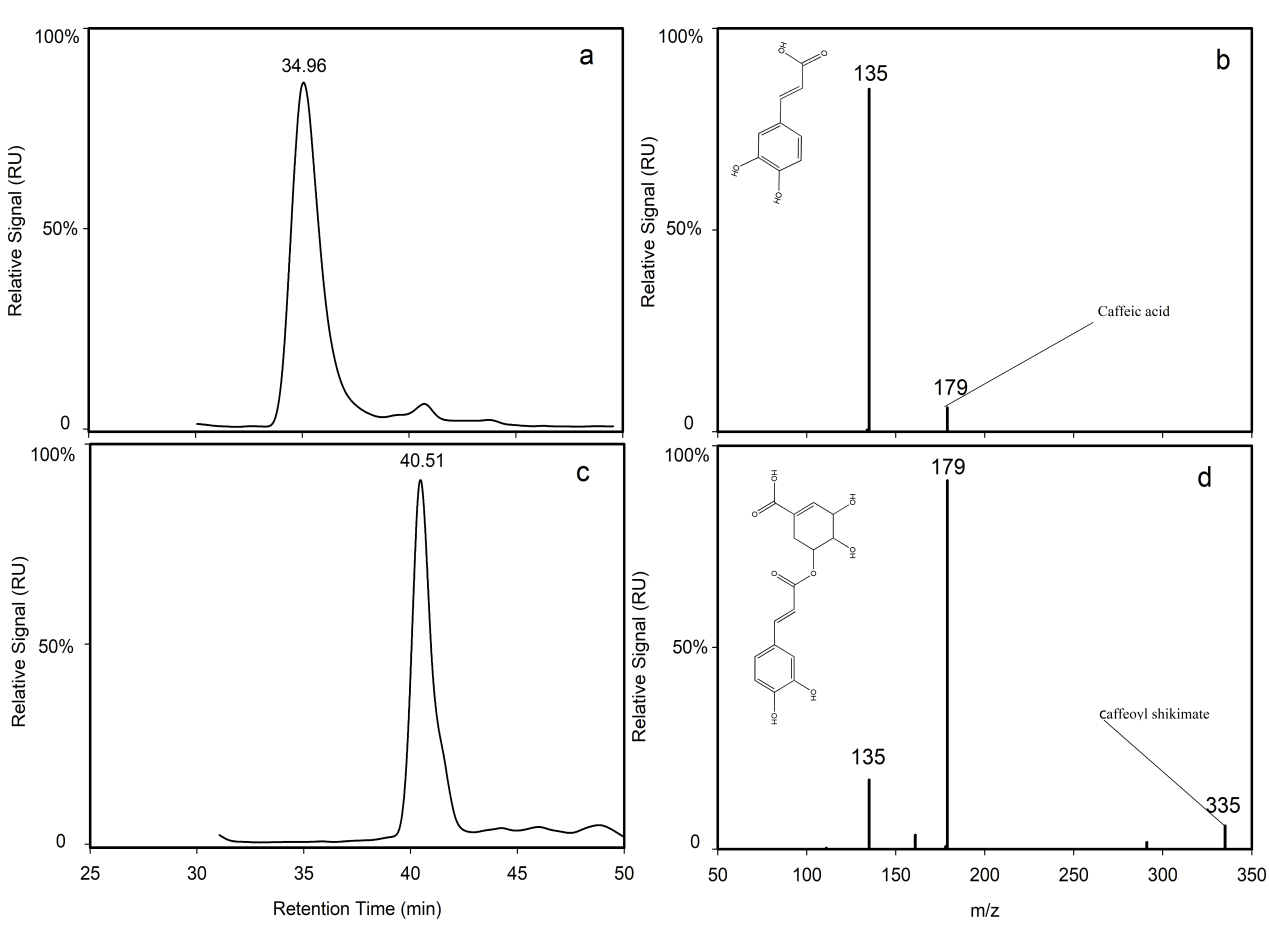


**Fig .S1** Standard samples of caffeoyl shikimate and caffeic acid. (a,b) caffeic acid; (c,d) caffeoyl shikimate.

**Table S4** *m/z* and retention time (RT) of caffeoyl shikimate and caffeic acid

| Name | *m/z* | Retention Time (min) |
| --- | --- | --- |
| caffeic acid | 179 | 34.96 |
| caffeoyl shikimate | 335 | 40.51 |
